# Optimization of Culture Conditions for Enrichment of *Saccharomyces cerevisiae* with Dl-α-Tocopherol by Response Surface Methodology

**Published:** 2017

**Authors:** Morteza Mohajeri Amiri, Mohammad Reza Fazeli, Mohsen Amini, Nasim Hayati Roodbari, Nasrin Samadi

**Affiliations:** a *Department of Biology, Science and Research Branch, Islamic Azad University, Tehran, Iran. *; b *Department of Drug and Food Control, Faculty of Pharmacy and Pharmaceutical Quality Assurance Research Center, Tehran University of Medical Sciences, Tehran, Iran. *; c *Department of Medicinal Chemistry, Faculty of Pharmacy and Drug Design and Development Research Center, Tehran University of Medical Sciences, Tehran, Iran.*

**Keywords:** Optimization, Enrichment, *Saccharomyces cerevisiae*, dl-α-tocopherol, Response Surface Methodology, Box-Behnken

## Abstract

Designing enriched probiotic supplements may have some advantages including protection of probiotic microorganism from oxidative destruction, improving enzyme activity of the gastrointestinal tract, and probably increasing half-life of micronutrient. In this study *Saccharomyces cerevisiae *enriched with dl-α-tocopherol was produced as an accumulator and transporter of a lipid soluble vitamin for the first time. By using one variable at the time screening studies, three independent variables were selected. Optimization of the level of dl-α-tocopherol entrapment in *S. cerevisiae* cells was performed by using Box-Behnken design via design expert software. A modified quadratic polynomial model appropriately fit the data. The convex shape of three-dimensional plots reveal that we could calculate the optimal point of the response in the range of parameters. The optimum points of independent parameters to maximize the response were dl-α-tocopherol initial concentration of 7625.82 µg/mL, sucrose concentration of 6.86 % w/v, and shaking speed of 137.70 rpm. Under these conditions, the maximum level of dl-α-tocopherol in dry cell weight of *S. cerevisiae* was 5.74 µg/g. The resemblance between the R-squared and adjusted R-squared and acceptable value of C.V% revealed acceptability and accuracy of the model.

## Introduction

Yeast enrichment technique that is used in food microbial studies aims at increasing the content of essential micronutrients such as vitamins and minerals in food supplements to compensate micronutrients deficiencies and to provide a public health benefit (1). *Saccharomyces cerevisiae, *known as baker’s yeast, is a well-known probiotic which can grow in simple environmental conditions and minimal media (2-5). Because of its safety and unique fermentation properties, yeast cells are commonly used in the human and animal foods and biotechnology industries (2, 6). European food safety authority (EFSA) categorized *S. cerevisiae*as as a microorganism that has QPS (Qualified Presumption of Safety) status (7). 

α-tocopherol (vitamin E) is a member of eight fat soluble compounds with a chromanol ring and tocopherol chain (8). It may protect cell membranes, low density lipoprotein, and human plasma from oxidative stress by scavenging free-radicals (9). Additionally, α-tocopherol probably has beneficial effects on pregnancy (10), age-related macular degeneration (11), cancer (12), cataracts (13), glaucoma (14), Alzheimer’s, and Parkinson’s diseases (15). Furthermore α-tocopherol is important in human and animals as immune modulator (16, 17). Fully synthetic α-tocopherol "dl-α-tocopherol" and its acetate derivative, which is more stable, are usually used in food supplements (18). 

There are a number of researches which have been used *S. cerevisiae* as a vehicle for some nutrients. Some studies reported uptake of essential trace metals such as zinc, iron, copper, and manganese by the yeast *S. cerevisiae *(1, 19 and 20). In addition to increasing the yeast biomass, the inorganic toxic metal elements with low bioavailability will be changed to safer and more bioactive types with the enhanced nutritional properties (21). Arshad *et al.* indicated that antioxidant activity of poultry meat was depended on the value of α-tocopherol concentration added to the diet (22). Furthermore α-tocopherol changed enzyme activity of the gastrointestinal epithelium in chicken which resulted in higher absorption of nutrients in digestive system (16). These studies indicated that using combination of α-tocopherol and *S. cerevisiae* may have some advantages including designing enriched probiotic supplements, protection of yeast as a probiotic microorganism from oxidative destruction, improving enzyme activity of the gastrointestinal tract and probably increasing half-life of α-tocopherol by protecting against destructor agents in digestive system.

Experimental design and statistical analysis are important tools for optimization (23). These techniques can be used for assessment of multivariable system with minimal experimental trials (23, 24). One of the most popular approach in experimental design is response surface methodology (RSM). RSM is a group of techniques for designing experiments, evaluating the interaction of variables, fitting appropriate mathematical models, and optimization of arbitrary responses (24).

In this study* S. cerevisiae* was used as a probiotic model for dl-α-tocopherol accumulation and transport for the first time. Three independent variables were selected for determination of optimal conditions for enrichment of *S. cerevisiae* biomass with dl-α-tocopherol by using statistical analysis and response surface methodology. 

## Experimental

*Microorganism, culture condition and chemicals*

*S. cerevisiae *ATCC 9763 was maintained on Sabouraud Dextrose agar (SDA, Merck Co. Germany) and stored at 4 °C. The dl-α-tocopherol was obtained from DSM Co. Basel, Switzerland. All other chemicals were obtained from Sigma-Aldrich Co. USA. 

*Fermentation conditions*

Shake flask experiments by using Sabouraud dextrose broth (SDB) were conducted to optimize cultivation conditions. The 500-mL Erlenmeyer flasks containing 200 mL of SDB medium were inoculated with 5% (v/v) of a 24-h preculture to give an initial yeast cell concentration of approximately equivalent to 0.1 g/L. Vitamin E (dl-α-tocopheryl acetate acetate) was added to medium at an initial concentrations of 2500-20000 µg/mL in preliminary studies. The cultures were incubated on a rotary shaker (J Labtech, DaihanLabtech Co.) at 100-200 rpm in dark condition. The samples were withdrawn at regular intervals and analyzed for determination of dry cell weight (g/L), and dl-α-tocopherol uptake (µg/g).

*Dry cell weight measurement*

For measurement of dry cell weight, 15 mL of SDB culture at each time interval was centrifuged at 8000×g for 10 min and washed twice with deionized water. The biomass was dried at 50 °C under reduced pressure and weighed for determination of dry cell weight.

*Determination of dl-α-tocopherol uptake*

*Extraction procedure*

Extraction of dl-α-tocopheryl acetate from the yeast cells was performed according to the method proposed by Popović* et al.* (25). The culture was centrifuged for 10 min at 1500×g and washed twice with phosphate-buffered saline (pH 7.4). Then, approximately 0.5 g of biomass was added to 30 mL of methanol kept at room temperature and dark condition for 16 h. The suspension was sonicated in an ultrasonic bath (Q Sonica, Q700) for 15-20 min. The solvent was evaporated at 40 °C under nitrogen steam. Then, 10 mL of n-hexan was added to the upper phase and sonicated for 15 min. Subsequently, n-hexan was evaporated. The residue was dissolved in 1 mL of n-hexan and filtered (0.22 μm pore size) prior to HPLC analysis.

*HPLC conditions*

Analysis was performed with Chemstation Software (Agilent Technologies). Chromatographic separation was performed on an Agilent column (Zorbax eclipse plus-C18 column), 10 cm, 2.1 mm, and 1.8 μm. Column oven temperature ranged from 25 to 40 °C. Mobile phase consisted of 100% n-hexan with a flow-rate of 0.3 mL/min and a pressure of 105 bars. The injection volume was 5 μL. Total run time was 20 min and experiment wavelength was 285 nm according to Popović *et al.* (25). The amount of uptaken dl-α-tocopherol was expressed as µg per gram of dry *S. cerevisiae* cells.

*Experimental Design and Statistical Analysis*

*Screening Study*

One-variable-at-a-time experiments were carried out to find the range of the parameters used for screening study. The levels of pH (4.5 to 7.5), temperature (25-35 °C), carbon source (glucose, sucrose, molasses, glycerol, maltose, starch, and fructose), nitrogen source (ammonium sulfate, meat extract, yeast extract, peptone and tryptone), primary vitamin E (dl-α-tocopheryl acetate) concentration, shaking speed, and incubation time were used in the optimization studies.

Our preliminary studies showed that among the selected factors, initial dl-α-tocopherol concentration, shaking speed and addition of sucrose to SDB medium play important roles in dl-α-tocopherol enrichment inside the yeast cells. 

*Optimization Design*

Optimum level of each parameter was determined by a statistical method termed response surface methodology (RSM) by using Design-Expert (version 10.0.1, Stat-Ease, Inc., Minneapolis, MN, USA). Optimization of the level of dl-α-tocopherol entrapment in *S. cerevisiae* cells was performed by Box-Behnken (26) as a RSM method. Variables that were considered significant in screening study, including initial dl-α-tocopherol concentration (A), shaking speed (B), and concentration of sucrose added to SDB medium (C), were defined at three levels (low, basal and high), coded as (-1, 0 and +1) (Table 1). In Box-Behnken technique designed by Design-Expert^® ^software, a set of seventeen experiments with five central point replicates was conducted.

The equation of the system was described by the modified quadratic polynomial model:

Y = *α*_0_ +∑*αiXi* + ∑*αiiXi*^2^ + ∑ *αijXiXj *+ *ε*

where Y is the response, *α*_0_ is a constant, *α*_i_ and *α*_ii_ reveal linear and quadratic effects respectively, *α*_ij_ is the quadratic effects of the interactions, *X*_i_ and *X*_j_ are coded values of the parameters and *ε* is the error of model. In this study Y reveals the expected dl-α-tocopherol amount in dry weight of yeast biomass. 

The significance of the model on the response was checked by the analysis of variance (ANOVA) and was determined by a *p*-value below 0.05. Goodness of fit for modified quadratic polynomial model equation was determined by the multiple correlation coefficient (R^2^) and adjusted R^2^. In this study, response surface plot indicating the effect of coded variable (dl-α-tocopherol concentration, shaking speed and sucrose) interactions and relations on dl-α-tocopherol amount in dry cell weight were main responses.

Plot of experimental versus predicted response values and plot of studentized residual versus predicted response values were checked.

## Results and Discussion

*Box-Behnken Design and experimental responses*

Our preliminary study showed that among selected factors, the dl-α-tocopherol concentration, shaking speed, and addition of sucrose to fermentation medium play important roles. 

The data obtained from experiments were analyzed statistically to determine which interaction was significant. According to Box-Behnken design, each parameter was changed at three levels while the other parameters were kept constant. The results of Box- Behnken design are shown in Table 2.

*Response Surface Methodology Analysis*

The information obtained from the design showed that the range of dl-α-tocopherol in dry weight was 1.48-5.64 (µg/g). To select the appropriate model to fit the data, the analysis of variance by calculating F-value was used (Table 3). According to the results, we found that a modified quadratic polynomial equation appropriately fit the data. The lack of fit F-value of 2.93 suggested that the lack of fit is not significant that presents acceptability of the model. 

**Figure 1:**
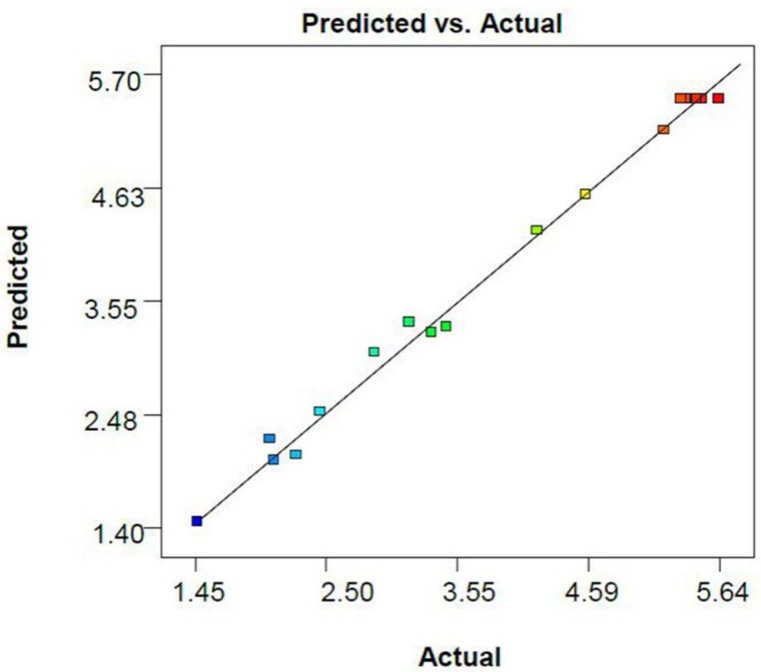
Plot of experimental versus predicted values of dl-α-tocopherol amounts per dry cell weight of *S. cerevisiae*

The R-squared (R^2^) and adjusted R-squared (R^2 ^adj) coefficients of this model are 0.993 and 0.986, respectively, indicating that the noise of the system is less than one percent. Furthermore, the resemblance between the R^2^ and adjusted R^2^ reveals the acceptability of the model to anticipate the results in the optimization. The predicted R^2^ (0.933) shows a very appropriate harmony between the value predicted by the model and the actual data (Figure 1).

Furthermore, the relative standard deviation (RSD) or C.V.% is 4.42 that indicates the accuracy and repeatability of the model. Adeq precision ratio of 32.833 indicates an adequate signal. Adeq precision measures the signal to noise ratio and a ratio of greater than 4 is desirable. It means that the model can be used to navigate the design space.

**Figure 2:**
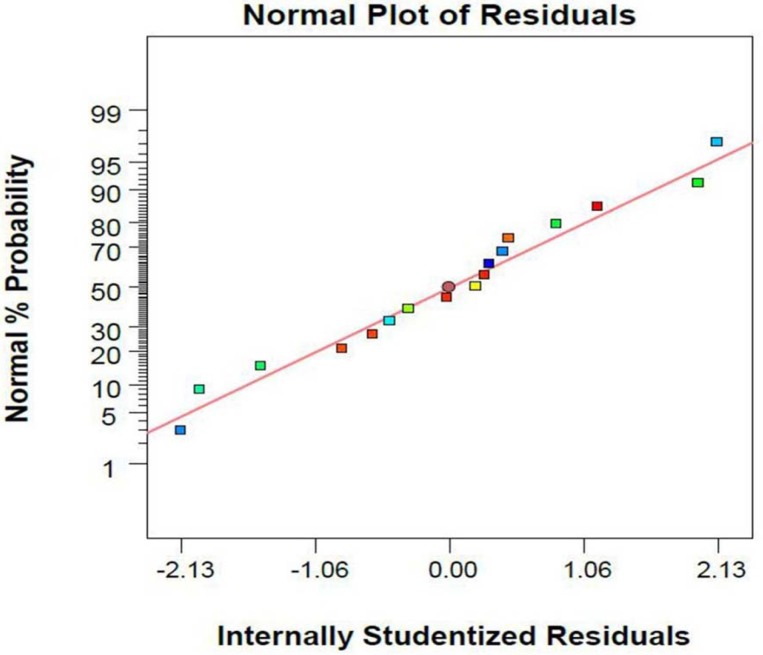
Plot of studentized residual versus predicted values of α-tocopherol amounts per dry cell weight of *S. cerevisia*

Absence of trends in the plot of studentized residual versus the values predicted by the model shows that the variances in the data are acceptable and no outliers are present in the experiments (Figure 2).

**Table 1:** Factors used in the Box-Behnken design and their levels

**Parameters**	**Unit**	**Symbol**	**Level -1**	**Level 0**	**Level +1**
α-tocopherol concentration	µg/mL	A	2500.0	6250.0	10000.0
Shaking speed	rpm	B	100.0	150.0	200.0
Sucrose	% w/v	C	2.0	5.0	8.0

**Table 2:** The Box-Behnken experiments and the dl-α-tocopherol amounts in dry cell weight of yeast.

**Response: dl-α-tocopherol per dry cell weight (µg/g)**	**Factor 3** **C: sucrose (% w/v)**	**Factor 2** **B: shaking speed (rpm)**	**Factor 1** **A: Initial dl-α-tocopherol con. (µg/mL)**	**Block**	**Run**	**Std**
2.2678	5.00	100.00	2500.00	Block1	2	1
4.1887	5.00	100.00	10000.00	Block1	1	2
1.4805	5.00	200.00	2500.00	Block1	9	3
2.0553	5.00	200.00	10000.00	Block1	17	4
2.4603	2.00	150.00	2500.00	Block1	8	5
3.4657	2.00	150.00	10000.00	Block1	16	6
2.8903	8.00	150.00	2500.00	Block1	4	7
5.2019	8.00	150.00	10000.00	Block1	12	8
3.1682	2.00	100.00	6250.00	Block1	5	9
2.0874	2.00	200.00	6250.00	Block1	10	10
4.5800	8.00	100.00	6250.00	Block1	6	11
3.3472	8.00	200.00	6250.00	Block1	3	12
5.5076	5.00	150.00	6250.00	Block1	13	13
5.4626	5.00	150.00	6250.00	Block1	15	14
5.6417	5.00	150.00	6250.00	Block1	14	15
5.3744	5.00	150.00	6250.00	Block1	7	16
5.3378	5.00	150.00	6250.00	Block1	11	17

**Table 3:** ANOVA for response surface modified quadratic model analysis of variance.

**Source**	**Sum of Squares**	**df**	**Mean Square**	**F-Value**	***p*** **-value Prob > F**
Model	33.46	8	4.18	148.35	< 0.0001 significant
A-Initialdl-α-tocopherol concentration	4.22	1	4.22	149.82	< 0.0001
B-shaking speed	3.42	1	3.42	121.49	< 0.0001
C-sucrose	2.93	1	2.93	103.78	< 0.0001
AB	0.45	1	0.45	16.07	0.0039
AC	0.43	1	0.43	15.13	0.0046
A2	8.01	1	8.01	284.01	< 0.0001
B2	10.62	1	10.62	376.55	< 0.0001
C2	1.42	1	1.42	50.47	0.0001
Residual	0.23	8	0.028		
Lack of Fit	0.17	4	0.042	2.93	0.1615 not significant
Pure Error	0.057	4	0.014		
Cor Total	33.68	16			
R^2^	0.9933				
R^2^ adj	0.9866				
C.V.%	4.42				
Adeq Precision	32.833				

**Table 4:** Validity of optimization design

**Response**	**Software prediction** **Value ** **(µg/g** **)**	**Validity experiment value ** **(µg/g** **)**	**95% CI low**	**95% CI high**
dl-α-tocopherol per dry cell weight	5.87	5.74	5.69	6.04

**Figure 3:**
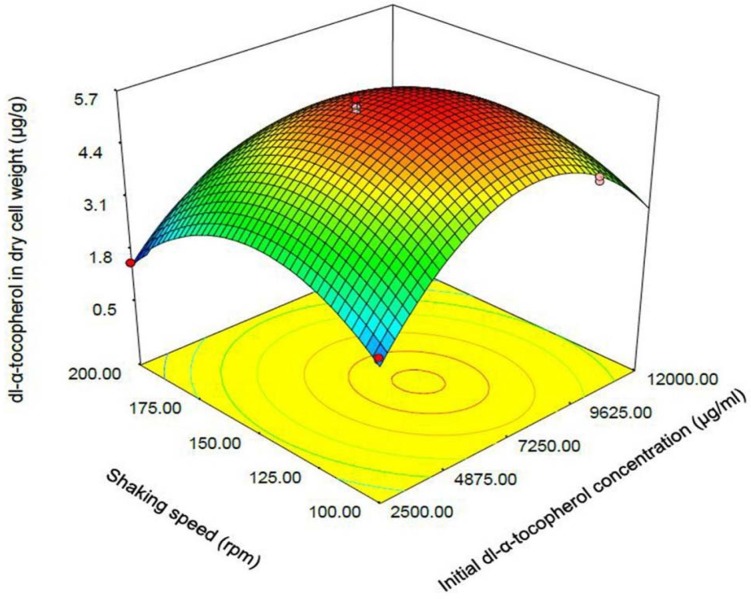
Response surface plot indicating the effect of shaking speed and initial dl-α-tocopherol concentration interaction on dl-α-tocopherol amount per dry cell weight of *S. cerevisia*

**Figure 4:**
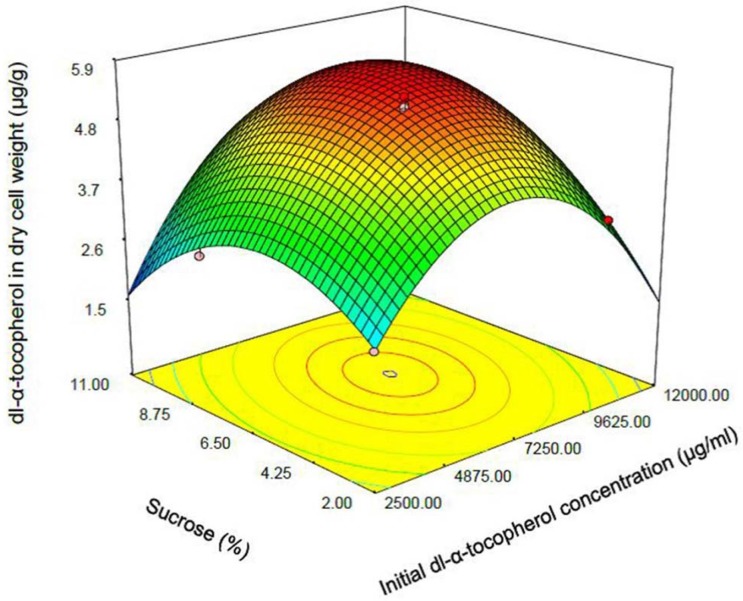
Response surface plot indicating the effect of sucrose (% w/v) added to culture medium and initial dl-α-tocopherol concentration interaction on dl-α-tocopherol amount per dry cell weight of *S. cerevisia*

**Figure 5:**
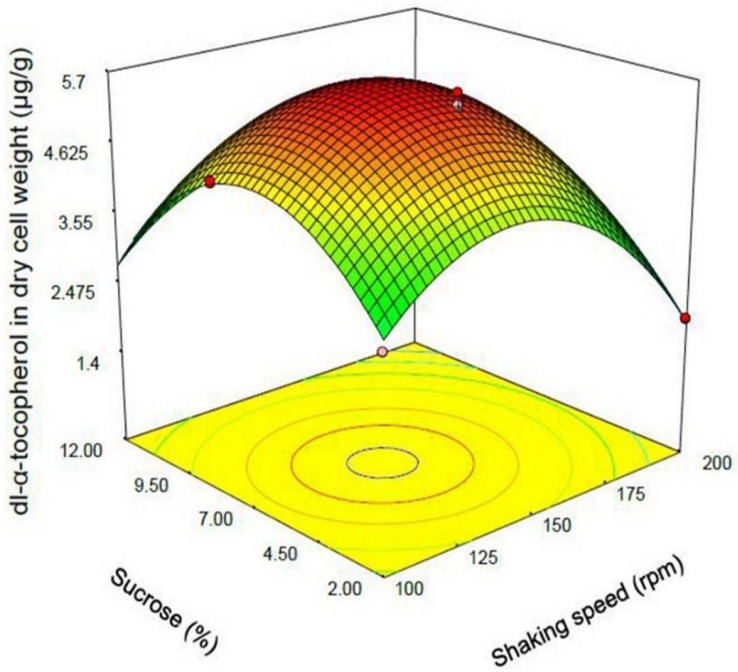
Response surface plot indicating the effect of sucrose (% w/v) added to culture medium and shaking speed (rpm) interaction on dl-α-tocopherol amount per dry cell weight of *S. cerevisia*

Eventually, in this study, the modified quadratic polynomial equation is an appropriate model to explain the level of dl-α-tocopherol amounts entrapped in *S. cerevisiae *cells. The regression analysis report for modified quadratic model showed that linear, squared, and interaction coefficients are significant (*p* < 0.05) except one linear interaction (Table 3).

The equation that software suggested to this experiment is as the follows:

Y = 5.46 + 0.73 A - 0.65 B + 0.60C - 0.34 AB + 0.33 AC- 1.38 A^2^ - 1.59 B^2^ - 0.58 C^2^

where Y is the dl-α-tocopherol amount in dry weight of yeast cells. A, B, and C are initial concentration of dl-α-tocopherol in culture medium, shaking speed, and concentration of sucrose added to SDB medium, respectively. The coefficients of A and C are positive that means positive effects of dl-α-tocopherol and sucrose concentrations on dl-α-tocopherol entrapment in *S. cerevisiae* cells. Negative coefficient of B means that the dl-α-tocopherol amount in dry cell weight of *S. cerevisiae *increases when shaking speed decreases.

The linear and quadratic coefficients of AB, A^2^, B^2^ and C^2^ are negative with *p* < 0.05, showing the significant influence of these factors. Interaction between dl-α-tocopherol concentration and shaking speed (AB) is more significant than interaction between dl-α-tocopherol concentration and sucrose (AC). The interaction between shaking speed and sucrose is insignificant (*p* > 0.05). Therefore, the modified quadratic polynomial model was used for optimization.

*Three-dimentional rosponce plots*

One of the most efficient tools in analysis of interactions is using graphical plots, especially three-dimensional response surface plot. In Figure 3, the relationship between shaking speed and initial dl-α-tocopherol concentrations investigated while the sucrose concentration was constant at the middle value. This plot indicates the quadratic coefficients of shaking speed and initial dl-α-tocopherol concentration is significant. The convex shape of three-dimentional plot reveals that we could calculate the optimal point of the response in the range of parameters. Figure 4 shows that when the sucrose and initial dl-α-tocopherol concentrations raised, the entrapment of dl-α-tocopherol in yeast cells increased and reached to an optimum level. The shape of plot reveals that the interaction between dl-α-tocopherol concentration and shaking speed is also significant. By raising the shaking speed and concentrations of sucrose and dl-α-tocopherol, the enriched cells of *S. cerevisiae* increased to an optimum point and then decreased. In Figure 5, the relationship between shaking speed and sucrose added to medium was investigated while the initial dl-α-tocopherol concentration was constant at the middle value. This plot shows that interaction between shaking speed and sucrose added to media is significant too. When the sucrose and shaking speed raised, the entrapment of dl-α-tocopherol in yeast cells increased and reached to an optimum level.

*Optimization value*

The optimum points of independent parameters to maximize the response were dl-α-tocopherol initial concentration of 7625.82 µg/mL, sucrose concentration of 6.86% w/v and shaking speed of 137.70 rpm. When the unstudied parameters were considered constant at the middle point, the maximum level of dl-α-tocopherol in biomass dry cell weight was predicted to be 5.866 µg/g. 

*Confirmation of the model*

For validation of the model, an experiment was performed with the variable values suggested by the software. By applying the optimum conditions, 5.74 µg of dl-α-tocopherol was extracted from each gram of dried yeast cells; approximately this was 97.8% of the value predicted by the modified quadratic model (Table 4). The observed values were in good agreement with the predicted value. These data confirm adequacy and significance of the model.

## Conclusions

This study is the first report about using *S. cerevisiae* as an accumulator and transporter of a lipid-soluble vitamin, dl-α-tocopherol. Our results showed that dl-α-tocopherol absorption in *S. cerevisiae* cells was affected significantly by shaking speed, sucrose, and dl-α-tocopherol concentrations. The modified quadratic model obtained from Design-Expert^® ^software was adequate (*p* < 0.001). The resemblance between the R-squared and adjusted R-squared and acceptable value of C.V% revealed acceptability and accuracy of model. Mechanisms that govern movement of this lipid-soluble substance along the plasma membrane of eukaryotic cells need further studies.
